# Noncommunicable disease burden among conflict-affected adults in Ukraine: A cross-sectional study of prevalence, risk factors, and effect of conflict on severity of disease and access to care

**DOI:** 10.1371/journal.pone.0231899

**Published:** 2020-04-21

**Authors:** Blanche Greene-Cramer, Aimee Summers, Barbara Lopes-Cardozo, Farah Husain, Alexia Couture, Oleg Bilukha

**Affiliations:** Emergency Response and Recovery Branch, Division of Global Health Protection, Centers for Disease Control and Prevention, Atlanta, Georgia, United States of America; University of Utah, UNITED STATES

## Abstract

**Background:**

There is limited research on noncommunicable diseases (NCDs) in humanitarian settings despite the overall global burden and disproportionate growth in many conflicts and disaster-prone settings. This study aimed to determine the prevalence of NCDs and assess the perceived effect of conflict on NCD severity and access to treatment among conflict-affected adults (≥ 30 years) in Ukraine.

**Methods and findings:**

We conducted two population-representative, stratified, cross-sectional household surveys: one among adult internally displaced people (IDPs) throughout Ukraine and one among adults living in Donbas in eastern Ukraine. One randomly selected adult per household answered questions about their demographics, height and weight, diagnosed NCDs, access to medications and healthcare since the conflict began, as well as questions assessing psychological distress, trauma exposure, and posttraumatic stress disorder. More than half of participants reported having at least one NCD (55.7% Donbas; 59.8% IDPs) A higher proportion of IDPs compared to adults in Donbas experienced serious psychological distress (29.9% vs. 18.7%), interruptions in care (9.7–14.3% vs. 23.1–51.3%), and interruptions in medication than adults in Donbas (14.9–45.6% vs. 30.2–77.5%). Factors associated with perceived worsening of disease included psychological distress (p: 0.002–0.043), displacement status (IDP vs. Donbas) (p: <0.001–0.011), interruptions in medication (p: 0.002–0.004), and inability to see a doctor at some point since the start of the conflict (p: <0.001–0.008).

**Conclusions:**

Our study found a high burden of NCDs among two conflict-affected populations in Ukraine and identified obstacles to accessing care and medication. Psychological distress, interruptions to care, and interruptions in medication were all reported by a higher proportion of IDPs than adults in Donbas. There is a need for targeted policies and programs to support the unique needs of displaced conflict-affected individuals in Ukraine that address the economic and perceived barriers to NCD treatment and care.

## Introduction

NCDs have received little attention in humanitarian settings worldwide despite their overall global burden [1,2]. A significant gap exists in understanding NCDs in emergency settings with few studies providing estimates of NCD burden in displaced populations or the effect of emergencies on access to care [1–12].

Prior to the start of the conflict in February 2014, Ukraine was a stable, middle-income country. Five years later, there are more than 4.4 million persons affected by the conflict [13]. In areas with active fighting, access to basic services such as healthcare, electricity, water, and food were interrupted, and in some places, never fully returned to pre-conflict stability [13]. Although Ukraine law indicates healthcare is free to all citizens, in practice most people have to cover many additional healthcare expenses from their own resources. Emigration of healthcare workers out of the conflict zone has caused reductions in care seeking because individuals could not see their preferred provider [13]. In 2018, less than two-thirds of IDPs were satisfied with the accessibility to healthcare [14]. Interruptions in access to care and medications can be particularly problematic for noncommunicable diseases (NCDs) requiring continuity of long-term care in order to effectively treat and manage the diseases.

To date, only a few studies have examined the effect of conflict and displacement on the health status and care seeking behaviors of Ukrainians [15–17]. An assessment of older persons (>60 years) conducted in the Donbas regions of Ukraine (an area that includes Donetsk and Luhansk regions) in early 2016 showed that approximately 70% of individuals suffered from at least one chronic disease, with hypertension and cardiovascular disease being the most prevalent. Additionally, the survey results showed a high prevalence of serious psychological distress ranging from 33% to 42% [15]. A cross-sectional study conducted between March and May 2016 found high prevalence of post-traumatic stress disorder (PTSD) (32%), depression (22%), and anxiety (17%) among adult (≥18 years) IDPs in Ukraine [16]. A high proportion of respondents likely needing mental health and psychosocial support reported not seeking it for reasons including inability to pay and not knowing where to find health services. A third study examined the prevalence of alcohol use disorder (AUD) among Ukrainian IDPs and found that AUD was present among men and alcohol users were significantly less likely to access mental health services than non-users [17].

This study aimed to add to this literature by comparing the prevalence of NCDs and associated risk factors among adults aged ≥ 30 years who were internally displaced across Ukraine or residing in government-controlled areas close to the conflict zone in eastern Ukraine (Donetsk and Luhansk regions) and examining the perceived effect of conflict on NCD severity and access to treatment in these populations.

## Methods

### Study design and sampling

We conducted two separate cross-sectional surveys among adult IDPs throughout Ukraine displaced due to conflict and among adults living in government-controlled, conflict-affected regions in Donbas regions in eastern Ukraine. For each survey we used an estimated prevalence for hypertension of 30% +/- 5% margin of error, with a 95% confidence interval and a DEFF of 2 to select a sample size of 667 rounded to 700 to ensure the minimum sample size was met. Additional methods for each survey are described separately below. To account for clustering from sampling, a mixed model was used with the cluster variable treated as a random effect in analysis.

### IDP survey

We conducted a population-representative, stratified, cross-sectional household survey of adults aged ≥30 years displaced due to conflict across Ukraine (excluding Donetsk & Luhansk regions). Individuals were eligible to participate if they were a current resident of a randomly selected IDP household and consented. Population data on the number of IDP households per region were obtained from the Ministry of Social Policy of Ukraine updated as of January 2018. According to the International Organization for Migration’s National Monitoring Survey, 95% of IDPs were registered [18]. The number of households selected per region (stratum) was based on a proportional distribution of registered IDPs in regions excluding Donetsk and Luhansk. Households within each region were selected through simple random sampling using government registration lists combined with a list of IDPs previously compiled by Kyiv International Institute of Sociology (KIIS), a research organization specializing in sociological research in Ukraine. These lists were not exhaustive, as some IDPs may remain unregistered. Lists were randomized, and households were contacted via telephone until the required number of consenting households per region was reached. Households were called three times before they were considered unreachable. Households reached by phone were considered eligible if they had at least one adult ≥30 years living there, were residing in survey area, had been or currently were displaced due to the conflict, and consented. These households were scheduled for an in-person interview.

### Donbas survey

We conducted a randomized cross-sectional two-stage household cluster survey of adults (≥30 years) living in government-controlled conflict-affected regions in eastern Ukraine (Donetsk and Luhansk regions). Individuals were eligible to participate if they were a current resident of a randomly selected cluster and household and consented. Population data for cluster selection were obtained from electoral statistical data from parliamentary elections in 2014. The primary sampling unit was the electoral precinct. Clusters were selected probability proportional to size, 21 clusters were allocated to Donetsk and eight clusters to Luhansk region, excluding those which were too close to the conflict line to collect data safely. Households for the second stage of sampling were systematically selected using a random starting point within each cluster. A skip pattern was followed that was adjusted depending on the type of housing (individual homes, small apartment buildings, large apartment buildings). Residences where no one was living were not included in the skip pattern.

### Procedures and questionnaire

One adult from each household was selected to participate using forms designed by KIIS. All eligible household members were listed in order of age and gender and then a participant was randomly selected based on which member was on the line that matched the number printed for that household. If the selected individual was unable to answer for themselves, psychological distress and trauma questions were not administered, but a caregiver answered demographic and chronic disease diagnoses questions. All participants were read a standardized informed consent form. Verbal consent was provided and was marked in a tracking diary before starting survey administration.

The survey included questions about the individual: age, sex, education, self-reported height and weight, and the household: current location, living situation, residence before displacement, length and number of displacements, number of people in household, income, access to non-governmental assistance. These questions, while not validated, were drawn from a previous study conducted in Ukraine to allow for comparability [15].

Participants were also asked if they had been diagnosed by a medical professional with any of the most common NCDs (hypertension, high cholesterol, cardiovascular disease, diabetes, cancer, kidney disease, and chronic lung disease), any other NCDs not covered in the previous categories, mental illness and overweight/obesity. For each reported diagnosis, participants were asked follow-up questions to capture date of diagnosis, perceived changes in disease severity since conflict, interruptions in medical care and access to NCD medication. Psychological distress was measured using the Kessler K6 Psychological Distress Scale [19], previously validated in Ukraine. PTSD and exposure to traumatic events were assessed using an adapted version of the Harvard Trauma Questionnaire, which combines the measurement of trauma events (part 1) and symptoms of PTSD (part 2) [20].

The questionnaire was translated into Russian and checked for accuracy by Russian-speaking Centers for Disease Control and Prevention (CDC) staff. The translated survey was reviewed and revised by local team members to improve phrasing of questions and clarity of language. Enumerators were trained on sampling procedures, interview techniques, and questionnaire administration. The study was determined to be non-research by the Centers for Disease Control and Prevention’s Institutional Review Board because the primary purpose of the study was to improve public health service for conflict-affected populations in Ukraine.

### Statistical methods

Data were double-entered into a customized database software by trained staff to ensure data quality. Analyses were conducted using STATA v15 (College Station, TX). The frequency of each indicator and p-values for the differences in prevalence were calculated using χ^2^ or Fishers exact tests. Continuous variables such as average household size, mean monthly household income, and mean household income per person were analyzed using 2-sample T-tests.

Bivariate and multivariate logistic regression were used to assess the association of potential risk factors with doctor diagnosed NCDs. The nine potential risk factors used as binary predictor variables were: study population (IDP vs. Donbass), age category (30–59 vs. 60+), sex, monthly household income (<5000 UAH [about 200 USD], ≥5,000 UAH), completed higher education, experienced serious psychological distress (>12 on Kessler scale), reported trauma exposure (none vs. any), meeting PTSD case criteria based on self-report, and presence of overweight or obesity (≥ 25kg/m^2^ vs <25 kg/m^2^) based on body mass index (BMI) calculated from self-reported height and weight. We assessed nine NCD diagnosis outcome variables: having any diagnosed NCD, hypertension (HTN), cardiovascular disease (CVD), high cholesterol (HCL), chronic lung disease (CLD), diabetes, cancer, mental illness (e.g. doctor diagnosed depression or anxiety), other NCD.

Bivariate and multivariate logistic regression were also used to assess the association of the risk factors with perceived worsening of disease. In addition to the nine potential risk factors listed above, we included in this analysis two additional binary variables: “experienced interruptions in medication since the conflict began” and “unable to see a provider for their NCD since the conflict began”. The eight outcome variables for this analysis were: perceived worsening since the conflict began of hypertension, cardiovascular disease, high cholesterol, chronic lung disease, diabetes, cancer, mental illness, or other NCD.

Each outcome variable was tested in bivariate analyses with each potential risk factor. Statistically significant risk factors at a *P* ≤ 0.05 level were included in a multivariate logistic regression model to estimate OR and 95% CI. In multivariate analysis we used logistic regression and backward elimination procedure to retain variables significant at *P* ≤ 0.05 level in the final model.

## Results

Both surveys were conducted May–June 2018. In Donbas, a total of 2093 households were visited. Of these, 725 (34.7%) households were present, had an adult ≥ 30 years old, consented to participate at the household level, and 696 of 725 (96.0% response rate) of adults randomly selected within these households agreed to be interviewed. Among IDPs, 3309 households were contacted by telephone and 1277 (38.5%) were eligible. Data were collected from adults in 704 eligible households resulting in a response rate of 55.1% (704/1277).

The demographic and socioeconomic characteristics of the survey participants are shown in Table 1. Just over two-thirds of participants were female, with a slightly higher proportion of women among IDPs. Median participant age was higher in Donbas than among IDPs. A higher proportion of IDPs had completed higher education or above compared to adults in Donbas. Nearly all participants in Donbas lived in a house or apartment they owned, while fewer than 5% of IDPs owned their current residence. IDPs reported higher monthly household income and monthly income per capita than those in Donbas. More than half of households in Donbas reported living on less than $200 per month. Two-thirds of IDPs received non-governmental assistance at some point since the conflict began compared to only 8.0% of those in Donbas. However, less than one-third of IDPs who ever received assistance reported receiving assistance in the last three months before the survey.

**Table 1:** Demographic and socioeconomic characteristics of conflict-affected adults in Ukraine, 2018.

	Donbas	IDPs*	*P* value
	**(N = 696)**	**(N = 704)**	
**Female (n, %)**	466 (67.0)	515 (73.2)	0.011
**Age (years) (n, %)**	**(N = 696)**	**(N = 704)**	<0.001
30–59	384 (55.2)	539 (76.6)	
≥ 60	312 (44.8)	165 (23.4)	
Mean (SD)	55.9 (15.5)	47.3 (13.7)	
Median (IQR)	57 (42–67)	43 (36–59)	
**Completed higher education or above (n, %)**	174 (25.0)	327 (46.5)	<0.001
**Current living situation (n, %)**			<0.001
Own house or apartment	642 (92.2)	31 (4.4)	
Living with relative, renting, social apartment^$^, other	54 (7.8)	673 (95.6)	
**Average household size (n, SD)**	2.2 (1.2)	2.9 (1.4)	<0.001
**Socioeconomic**			
**Total # HH members with money-earning job (n, %)**	**(N = 696)**	**(N = 704)**	<0.001
0	324 (46.6)	218 (31.0)	
≥1	372 (53.5)	486 (69.0)	
**Monthly household income (UAH) (n, %)**	**(N = 562)**	**(N = 590)**	<0.001
<5000	326 (58.0)	189 (32.0)	
≥5000	236 (42.0)	401 (68.0)	
Mean(USD) (SD)	$169 (110)	$268 (187)	
**Mean monthly household income per person (USD) (SD)**	$87 (47)	$116 (113)	<0.001
**Non-governmental Assistance**			
	**N = 696**	**N = 704**	
**Ever received non-governmental assistance (n, %)**	51 (8.0)	486 (69.0)	<0.001
	**N = 51**	**N = 486**	
**Received non-governmental assistance in past 3 months**^#^	15 (29.4)	140 (28.8)	0.928
**Obesity and Mental Health**			
	**N = 696**	**N = 704**	
**Overweight or Obese (n, %)**	137 (20.0)	104 (15.0)	<0.001
	**N = 680**	**N = 692**	
**Trauma exposure**^&^ **(n, %)**	268 (39.4)	649 (93.8)	<0.001
**Serious psychological distress**^+^ **(n, %)**	127 (18.7)	207 (29.9)	<0.001
**Post traumatic stress disorder (n, %)**	1 (0.2)	17 (2.5)	<0.001

*IDP—internally displaced person

^$^Social apartment—apartment subsidized by government funding

^#^Of those who had ever received assistance

^&^Trauma exposure: any reported exposure

^**+**^ Experienced serious psychological distress: scoring >12 on the Kessler (compared to those scoring ≤12)

The prevalence of overweight or obesity (based on self-reported weight and height) was significantly higher in Donbas than among IDPs. A higher proportion of IDPs reported trauma exposure and experiencing significant psychological distress and PTSD compared to adults in Donbas (Table 1). We found that significant psychological distress was statistically significantly higher among women than men in Donbas (p<0.007) and among IDPs (p<0.0002).

The prevalence of NCD diagnosis by study area, age and sex is presented in Table 2. More than half of participants reported at least one diagnosed NCD. The most prevalent NCDs were hypertension and other cardiovascular diseases. Approximately 7% of participants reported diabetes, and 3–5% reported cancer diagnosis. Generally, prevalence of NCD diagnosis was higher in older age group, and in females compared to males.

**Table 2:** Noncommunicable disease prevalence among conflict-affected adults in Ukraine, by sex and age.

	Donbas					IDPs*				
	Overall (N = 696)	Male (N = 230)	Female (N = 466)	30–59 yrs (N = 384)	60+ yrs (N = 312)	Overall (N = 704)	Male (N = 189)	Female (N = 515)	30–59 yrs (N = 539)	60+ yrs (N = 165)
Any	388 (55.7)	102 (44.4)	286 (61.4)	147 (38.3)	241 (77.2)	421 (59.8)	99 (52.4)	322 (62.5)	266 (49.4)	155 (93.9)
Hypertension	267 (38.4)	56 (24.4)	211 (45.3)	81 (21.1)	186 (59.6)	196 (27.8)	41 (21.7)	155 (30.1)	93 (17.3)	103 (62.4)
Cardiovascular	166 (23.9)	38 (16.5)	128 (27.5)	39 (10.2)	127 (40.7)	176 (25.0)	42 (22.2)	134 (26.0)	82 (15.2)	94 (57.0)
High cholesterol	50 (7.2)	9 (3.9)	41 (8.8)	11 (2.9)	39 (12.5)	84 (11.9)	18 (9.5)	66 (12.8)	35 (6.5)	49 (29.7)
Diabetes	49 (7.0)	10 (4.4)	39 (8.4)	12 (3.1)	37 (11.9)	52 (7.4)	13 (6.9)	39 (7.6)	18 (3.3)	34 (20.6)
Chronic lung	29 (4.2)	8 (3.5)	21 (4.5)	13 (3.4)	16 (5.1)	39 (5.5)	12 (6.4)	27 (5.2)	22 (4.1)	17 (10.3)
Cancer	19 (2.7)	5 (2.2)	14 (3.0)	6 (1.6)	13 (4.2)	32 (4.6)	1 (0.5)	31 (6.0)	15 (2.8)	17 (10.3)
Mental illness	7 (1.0)	3 (1.3)	4 (0.9)	4 (1.0)	3 (1.0)	28 (4.0)	5 (2.6)	23 (4.5)	21 (3.9)	7 (4.2)
Other	269 (24.3)	38 (16.5)	131 (28.1)	67 (17.5)	102 (32.7)	296 (42.1)	65 (34.4)	86 (16.7)	189 (35.1)	107 (64.9)

*IDP—internally displaced person

In the multivariate analysis, age was significantly associated with increased risk of diagnosis for all NCD outcomes except for mental illness and chronic lung disease (Table 3). Serious psychological distress was also significantly associated with increased risk of all NCD outcomes except for diabetes. Female sex was associated with increased risk of any NCD, hypertension, cancer, and other NCDs. Overweight and obesity was associated with increased risk of any NCD, hypertension, cardiovascular disease, diabetes and high cholesterol. IDP (vs. Donbas) population status was associated with increased risk for high cholesterol, mental illness and other NCDs. Education and PTSD status were not significantly associated with NCD outcomes (Table 3).

**Table 3:** Risk factors associated in multivariate logistic regression with NCD diagnoses among conflict-affected adults in Ukraine, 2018.

Outcome	Predictor	aOR	95% CI	*P* value
Any NCD (N = 1,372)	≥60 years old	5.75	4.26, 7.76	<0.001
	Female	1.72	1.32, 2.24	<0.001
	Experienced serious psychological distress^+^	3.37	2.44, 4.63	<0.001
	Trauma exposure^&^	1.95	4.49, 2.55	<0.001
	Overweight	2.11	1.64, 2.70	<0.001
Hypertension (N = 1,372)	≥60 years old	4.86	3.61, 6.49	<0.001
	Female	1.88	1.38, 2.56	<0.001
	Income ≥ 5000 UAH	0.62	0.47, 0.83	0.001
	Experienced serious psychological distress^+^	2.68	1.96, 3.67	<0.001
	Overweight	4.15	3.07, 5.62	<0.001
Cardiovascular disease (N = 1,372)	≥60 years old	5.22	3.87, 7.05	<0.001
	Experienced serious psychological distress^+^	3.17	2.34, 4.29	<0.001
	Overweight	2.12	1.55, 2.89	<0.001
High cholesterol (N = 1,372)	IDP*	2.64	1.75, 4.00	<0.001
	≥60 years old	4.06	2.68, 6.14	<0.001
	Experienced serious psychological distress^+^	2.33	1.58, 3.45	<0.001
	Overweight	2.33	1.48, 3.68	<0.001
Chronic lung disease (N = 1,372)	Income ≥ 5000 UAH	0.56	0.33, 0.96	0.035
	Experienced serious psychological distress^+^	1.85	1.09, 3.14	0.024
Diabetes (N = 1,372)	≥60 years old	4.56	2.83, 7.35	<0.001
	Overweight	3.09	1.73, 5.49	<0.001
Cancer (N = 1,372)	≥60 years old	2.83	1.57, 5.11	0.001
	Female	2.65	1.11, 6.36	0.029
	Experienced serious psychological distress^+^	1.99	1.10, 3.59	0.022
	Trauma exposure^&^	2.92	1.34, 6.37	<0.001
	IDP*	3.65	1.48, 8.98	0.005
Mental illness (N = 1,372)	Experienced serious psychological distress^+^	5.65	2.69, 11.85	<0.001
	IDP*	2.20	1.16, 4.17	0.016
Other (N = 1,372)	≥60 years old	2.47	1.90, 3.22	<0.001
	Female	1.63	1.24, 2.15	0.001
	Experienced serious psychological distress^+^	1.84	1.40, 2.41	<0.001
	Trauma exposure^&^	1.54	1.10, 2.16	0.012

^&^Trauma exposure: any reported exposure

^**+**^ Experienced serious psychological distress: scoring >12 on the Kessler (compared to those scoring ≤12)

*IDP—internally displaced person

Between one-quarter and three-quarters of adults with an NCD diagnosed prior to the conflict reported a perceived worsening in the severity of their NCD since the start of the conflict (Table 4). Nearly double the proportion of IDPs reported perceived worsening of their disease compared to participants in Donbas. The proportion of participants reporting interruptions in care were ranged from nearly two (diabetes and mental illness) to four (hypertension, cardiovascular disease, cancer) higher among IDPs compared to adults with NCDs in Donbas. Overall, a higher proportion of IDPs with NCDs reported experiencing interruptions in access to their NCD medication compared to those in Donbas.

**Table 4:** Proportion of conflict-affected adults reporting perceived worsening of noncommunicable disease, experienced interruptions in care, and experienced interruptions in medication, Ukraine, 2018.

	Donbas			IDPs*		
	Perceived worsening	Care interruptions	Medication interruptions^+^	Perceived worsening	Care interruptions	Medication interruptions^&^
Hypertension	41.2	9.7	31.4	71.4	41.3	77.5
Cardiovascular disease	41.6	11.4	31.8	65.9	47.2	54.2
High cholesterol	22.0	12.0	44.2	57.1	39.3	65.8
Chronic lung disease	20.7	13.8	29.6	64.1	51.3	56.4
Diabetes	38.8	14.3	14.9	61.5	23.1	30.2
Cancer	31.6	10.5	38.5	59.4	50.0	55.0
Mental illness	28.6	14.3	42.9	67.9	32.1	46.4
Other	34.9	13.6	45.6	63.2	40.9	52.7

*IDP—internally displaced person

^**+**^The sample sizes for medication interruptions for Donbas were as follows: hypertension (N = 255), cardiovascular disease (N = 157), high cholesterol (N = 43), chronic lung disease (N = 27), diabetes (N = 47), cancer (N = 13), mental illness (N = 6), other (N = 134)

^&^The sample sizes for medication interruptions among IDPs were as follows: hypertension (N = 182), cardiovascular disease (N = 166), high cholesterol (N = 73), chronic lung disease (N = 32), diabetes (N = 43), cancer (N = 20), mental illness (N = 21), other (N = 254)

Self-reported increases in stress and anxiety since conflict onset were the most frequently reported reasons for perceived worsening of NCDs ranging from 33.9% to 100% of responses. Natural progression of the disease (up to 41.1% in Donbas and 26.3% of IDPs) and lack of income (up to 27.8% in Donbas and 21.1% of IDPs) were the other most common reasons reported. Cost was the main reason adults in Donbas and IDPs reported they were unable to see a doctor for their NCD since the conflict began, mentioned by up to 54.5% of adults with NCDs in Donbas and 71.9% of IDPs. Cost was also the main reported reason for interruptions in NCD medication: 25.0–100% of adults with NCDs in Donbas and 68.2–87.5% of IDPs, depending on the NCD.

In multivariate analysis, IDP (vs. Donbas) population status was significantly associated with perceived worsening of diabetes, high cholesterol, chronic lung disease and other NCDs (Table 5). Serious psychological distress was also significantly associated with perceived worsening of hypertension, mental illness and other NCDs. Interruption in access to medication was significantly associated with perceived worsening of cardiovascular disease, diabetes and high cholesterol. Interruption of access to medical care was associated with perceived worsening of hypertension and other NCDs. Neither age nor sex were significantly associated with perceived worsening of NCD (Table 5).

**Table 5:** Risk factors associated in multivariate analysis with perceived worsening of noncommunicable disease.

Outcome	Predictor	aOR	95% CI	*P* value
Perceived worsening of hypertension (N = 454)	Experienced serious psychological distress^+^	2.01	1.29, 3.13	0.002
	Trauma exposure^&^	2.11	1.25, 3.55	0.005
	Unable to see doctor	2.29	1.29, 4.04	0.004
Perceived worsening of cardiovascular disease (N = 315)	Interruptions in medication	2.39	1.44, 3.97	<0.001
Perceived worsening of high cholesterol (N = 113)	IDP*	4.93	1.93, 12.62	0.001
	Interruptions in medication	3.99	1.66, 9.61	0.002
Perceived worsening of chronic lung disease (N = 65)	IDP*	7.5	2.32, 24.27	0.001
Perceived worsening of diabetes (N = 88)	IDP*	3.39	1.33, 8.65	0.011
	Interruptions in medication	7.29	1.88, 28.31	0.004
Perceived worsening of cancer (N = 50)	Completed higher education	3.55	0.99, 1.90	0.057
Perceived worsening of mental illness (N = 33)	Completed higher education	9.39	0.99, 89.23	0.051
	Experienced serious psychological distress^+^	6.51	0.95, 44.67	0.056
Perceived worsening of other NCD (N = 457)	IDP*	2.17	1.36, 3.47	0.001
	Experienced serious psychological distress^+^	1.81	1.13, 2.92	0.014
	Unable to see doctor	3.26	1.95, 5.44	<0.001

^**+**^ Experienced serious psychological distress: scoring >12 on the Kessler (compared to those scoring ≤12)

^&^Trauma exposure: any reported exposure

*IDP—internally displaced person

## Discussion

This study found that more than half of conflict-affected adults (≥ 30 years) in Ukraine reported having at least one doctor-diagnosed NCD, higher than prevalence found for neighboring Poland and approaching prevalence levels in Western European countries like Germany and Austria [21]. Nationally, NCDs account for 91% of all deaths in Ukraine, national disease prevalence estimates are only available for three NCDs: diabetes (9% in 2014), hypertension (32% in 2015), and obesity (26% in 2016) [22]. While the prevalence of diabetes and hypertension found in our study were similar to the national estimates, the prevalence of overweight and obesity (20% in Donbas, 15% among IDPs) was lower than the national estimate. Compared to non-camp based Syrian refugees in Jordan and Lebanon, displaced Ukrainians had higher prevalence of both hypertension and diabetes (Jordan: 11% hypertension, 6% diabetes; Lebanon 7% hypertension, 3% diabetes)and similar prevalence of diabetes (Jordan: 7% vs. 6%) [23,24].

As expected, age was significantly associated with all NCD outcomes except mental illness and chronic lung disease in the multivariate analysis. Our study found higher prevalence of older adults diagnosed with at least one NCD (78% Donbas and 95% IDPs) compared with those found in the 2016 study of older persons in eastern Ukraine (68% and 74% in the government controlled and non-government controlled areas respectively) [15]. The variance in findings may be driven by the difference in study populations and the higher prevalence of NCDs among displaced adults outside of Donbas. Alternatively, the variance could also reflect a worsening of conditions over time due to the conflict. While a higher proportion of IDPs reported having any NCD, being displaced was only a risk factor for cancer, cholesterol, and mental illness after adjusting for age.

Our study found that a higher proportion of women than men reported all diagnosed NCDs, except for mental illness in Donbas. While overweight status may explain some of the difference in NCD prevalence between sexes (the study found more women were overweight than men), after controlling for overweight status in the multivariate analysis, women still reported a higher prevalence of having any NCD, hypertension, cancer, and other NCDs. Women affected by conflict in Ukraine may have experienced more stress and anxiety related to the conflict, or experience the stress more acutely, which may worsen the disease experience prompting them to seek treatment [25–27]. In our survey, we also found that psychological distress was higher among women than men in Donbas and among IDPs. Interestingly, the main factors associated with NCD prevalence (age, sex, and overweight) were not significantly associated with perceived worsening of NCDs. While displacement status (being an IDP vs living in Donbas) was significantly associated only with prevalence of high cholesterol, mental illness, and other NCDs it was significantly associated with perceived worsening of high cholesterol, chronic lung disease, diabetes, and other NCDs even after controlling for interruptions in care and medication. Perceived worsening may be tied to lack of social support with displaced persons feeling more isolated having left their communities and, sometimes, family members behind. While lack of social support has not been studied in relationship to perceived worsening of NCDs, it has been linked with other negative health outcomes such as increased mortality and hospital admissions in other European countries [28–30].

Of the three mental health outcomes included in the study, experiencing serious psychological distress was the most frequently associated with NCD prevalence and perceived worsening. Psychological distress was associated with prevalence of all NCDs, except diabetes, and with perceived worsening of hypertension, mental illness, and other NCDs, while trauma exposure was only associated with prevalence of any NCD, cancer, other NCDs and perceived worsening of hypertension. This may suggest that exposure to trauma does not always result in the same level of psychological distress among the respondents, and therefore also lead to different associations and perceptions of NCD severity. The growing body of literature looking at NCD and mental illness comorbidities has shown that mental distress may lead to unhealthy coping behaviors, such as drinking, smoking, and sedentary behavior, that are known risk factors for NCDs [17, 31]. While further research is needed to understand this relationship in conflict-affected and displaced populations, the association between psychological distress and perceived worsening of NCDs also highlights the need to train general practitioners to identify and address comorbid mental illnesses or strengthen referral chains in settings where populations may experience trauma, particularly when displaced from familiar surroundings and care providers. Mental health specialists and social service workers should be integrated into local and district clinics and hospitals as well as those in major urban hubs where many IDPs relocate. Every facility should be able to provide integrated care for NCDs and mental illness together, reducing the burden on the patient and the treatment gap that exists with inefficient referral processes.

Being displaced, experiencing serious psychological distress, interruptions in medication, and being unable to see a doctor at any point since the conflict started were the factors most frequently associated with perceived worsening of disease among both study populations. Participants reported that cost was the most common reason they were unable to see a doctor (52.2% Donbas, 69.7% IDPs) or experienced an interruption in their medication (25.0–100% in Donbas and 68.2–87.5% in IDPs depending on the NCD), supporting the finding by Roberts et al that cost plays a significant role in access to care and medication among displaced populations [16]. While healthcare in Ukraine is free for Ukrainians, our findings likely reflect costs associated with specific NCD medications or transport to appointments and pharmacy rather than the cost of healthcare itself. These findings suggest a need to further subsidize the cost of services and medication available to conflict-affected and displaced populations to reduce barriers to seeking care. Participants in our study who were currently displaced at the time of interview also reported challenges in seeing their preferred doctors where they were registered at their old residence and going to their preferred facilities affecting their continuity of care post-conflict, a point echoed among Syrian refugees in Jordan [32]. This highlights the need not just for service provision, but also educational programming and outreach about services available for displaced and conflict affected populations and where to go to connect with different services. Additionally, general practitioners and other medical staff should be trained to provide culturally adapted “survival kits” to facilitate self-care among displaced or migrating populations to help reduce gaps in care and treatment by providing a bridge until NCD patients can connect with appropriate care in their new location.

The findings of this study are subject to several limitations. While we used registration lists for IDPs, these were non-exhaustive and did not include households who were unregistered, without a working phone number, or were unavailable during the survey period. However, these lists were the best available at the time and used in previous studies [15]. Unregistered IDPs may be less likely to receive benefits and assistance and may have a lower socio-economic status, factors which may increase their risk of NCDs [4,33]. The study also had a high non-response rate among eligible IDP households. Households and individuals who chose to participate may have been those less traumatized or less affected by the conflict. The opposite may be true as well, that those willing to participate were those most affected and were trying to find help or more assistance. There were also some conflict-affected areas, such as the non-government-controlled areas, that were not included in the survey due to safety concerns for data collectors. Individuals in these areas may have worse health outcomes and access to care than survey participants. The numbers of persons who reported worsening of certain NCD conditions such as mental illness and chronic lung disease were small, therefore their findings should be interpreted with caution. BMI calculations were based on self-reported height and weight, which may underestimate the prevalence of overweight and obesity. Lastly, our study only captured previously diagnosed disease, it is likely an underestimate of the true prevalence of NCDs because it did not capture undiagnosed disease. Lastly, we did not collect information on substance use and any changes in use behavior following the conflict, which is a limitation given their link with accessing health services found in Ramachandran et al [17].

## Conclusion

Our study found more than half of participants had at least one NCD and the prevalence of hypertension, cardiovascular disease, and high cholesterol were similar to those in some Western European countries. Challenges in continuity of care and medication access after the start of the conflict were frequently reported, suggesting a need for integration of NCD programming (including mental health services) into primary care and targeted intervention planning in future response efforts. Programs to address the high cost of both health care and medication need to be developed and aligned with the government’s declaration of free health care and medications for displaced populations. Participants may have challenges connecting with the designated services, so there needs to be additional focus on raising public awareness and connecting individuals with the network of support services available. Similar support (related to cost or connecting with care networks) needs to be provided for non-displaced, conflict-affected populations, such as those in Donbas, who reported related challenges.

This study adds to the existing literature by describing the burden of NCDs and mental illness among conflict-affected individuals in two types of settings, internally displaced and those still living in the conflict area. In addition, this is first known study that explored the perceived effect of conflict on NCD severity and access to care among those affected. Future studies should look at the potential relationship between perceived worsening and its association with disease status/severity and mental wellbeing. Perception of disease severity may influence health (physical and mental) status, care seeking behaviors and unhealthy coping behaviors in conflict-affected populations and may be important for organizations to include when developing interventions and programs in these settings.

## Supporting information

S1 Data (XLSX)

S1 Questionaire (DOCX)

S2 Questionaire (DOCX)
