# Supplementary material for: Noncommunicable disease burden among conflict-affected adults in Ukraine: A cross-sectional study of prevalence, risk factors, and effect of conflict on severity of disease and access to care
Source: PLoS One. 2020 Apr 21;15(4):e0231899. doi: 10.1371/journal.pone.0231899 (PMC7173772; doi:10.1371/journal.pone.0231899)
Supplement: S1 Questionaire — (DOCX) [file pone.0231899.s002.docx]

**Assessment of Burden of NCDs in Populations Affected by Armed Conflict in Eastern Ukraine**

**Date of Survey**

**Interviewer Code**

1 8

**MM**

**YY**

**DD**

**HH**

**MM**

**Time of beginning:**

**Oblast**

**Cluster**

**Household**

**Number**

**Number of eligible**

**people in household**

**(> 30 years and currently home)**

**Respondent’s Relationship to Person >=30**

1 = Self

2 = Spouse

3 = Son/Daughter/

Grandson/Granddaughter

4 = Other (Specify)

**Type of settlement**

1 = Oblast center

2 = Urban

3 = Rural town

4 = Rural village

**Birth date of person >=30**

**DD**

**MM**

**YYYY**

**Age of person >=30 (complete years)**

**Sex of person >=30**

1 = Male
2 = Female

**Section A: Household Information**

*Instructions: SAY:* ***“Now I will ask you questions about your education and the household in which you are currently living.”***

| 1. | What is your education level? | 1 = Incomplete secondary school  2 = Complete secondary school  3 = Professional secondary education (technikum, uchilische)  4 = Incomplete higher education  5 = Complete higher education or above  9 = Don’t know |  |
| --- | --- | --- | --- |
| 2. | In what type of housing do you currently live? | 1 = House or apartment you own (no rent fee)  2 = Living with relatives or friends (no rent fee)  4 = Social apartments (pay for utilities but rent is free)  5 = Other |  |
| 3. | a. Were you ever displaced from your permanent residence due to the conflict since 2014? | 0 = No (**Skip to Q4**)  1 = Yes |  |
|  | b. Are you living in the same residence as you were in 2013/before the war? | 0 = No  1 = Yes (**Skip to Q3d**) |  |
|  | c. Where was your permanent residence located before the conflict? | 1 = Donetsk city  2 = Luhansk city  3 = Donetsk Oblast  4 = Luhansk Oblast  5 = Crimea  6 = Other |  |
|  | d. How long were you displaced?  *(Write “9” if don’t know)* | Write total number of months | |
| 4. | What is the total number of people, including you currently living in the household? | Write number of people |  |
| 5. | How many people in the household currently have a money-earning job? | Write number.  (Write “9” if don’t know) |  |
| 6. | What was the total monthly income of the household last month (Hrn)? | Write income amount.  (Write “9999” if don’t know) |  |

**Section B: Assistance**

*Instructions: SAY* ***“Now I will ask you what types of non-government assistance you or your household received due to the conflict since it began in February 2014.”***

| 1. | Have you or your household received any type of non-government assistance due to the conflict since it began in February 2014? | 0 = No (**Skip to Section C**)  1 = Yes  9 = Don’t know (**Skip to Section C**) |  |
| --- | --- | --- | --- |
| 2. | a. Cash vouchers or bank cards (not stipends or pensions from the government)  *If yes,*  b. How many times in the last 3 months did you receive this assistance? | 0 = No (**Skip to 3**)  1 = Yes  9 = Don’t know (**Skip to 3**)  0 = none/no times  1 = 1 time  2 = 2 times  3 = 3 times  4 = >3 times  9 = Don’t know |  |
| 3. | a. Non-government medical assistance (medications, clinician visits, transportation to clinic)  *If yes,*  b. How many times in the last 3 months did you receive this assistance? | 0 = No (**Skip to 4)**  1 = Yes  9 = Don’t know (**Skip to 4**)  0 = none/no times  1 = 1 time  2 = 2 times  3 = 3 times  4 = >3 times  9 = Don’t know |  |
| 4. | a. Food assistance  *If yes,*  b. How many times in the last 3 months did you receive this assistance? | 0 = No (**Skip to 5)**  1 = Yes  9 = Don’t know (**Skip to 5**)  0 = none/no times  1 = 1 time  2 = 2 times  3 = 3 times  4 = >3 times  9 = Don’t know |  |
| 5. | a. Non-food assistance not from government (hygiene items, detergents, blankets, household items, etc.)  *If yes,*  b. How many times in the last 3 months did you receive this assistance? | 0 = No (**Skip to 6)**  1 = Yes  9 = Don’t know (**Skip to 6**)  0 = none/no times  1 = 1 time  2 = 2 times  3 = 3 times  4 = >3 times  9 = Don’t know |  |
| 6. | a. Non-government transportation assistance (to go shopping, to government offices, to collect food, but not to the clinic)  *If yes,*  b. How many times in the last 3 months did you receive this assistance? | 0 = No (**Skip to Section C)**  1 = Yes  9 = Don’t know (**Skip to Section C**)  0 = none/no times  1 = 1 time  2 = 2 times  3 = 3 times  4 = >3 times  9 = Don’t know |  |

**Section C: Health Information**

*Instructions: SAY:* ***“Now I will ask you questions about your health issues and medications that you are taking.”***

1. Hypertension

| 1. | Have you been diagnosed with hypertension? | 0 = No (**Skip to next section C2)**  1 = Yes |  |
| --- | --- | --- | --- |
| 1b. | When were you diagnosed?  *Ask the participant to give you the date they were diagnosed. If Feb 2014 or before, select before. If after Feb 2014, select after.* | 0 = Before  1 = After |  |
| 1c. | Has your hypertension become worse, stayed the same, or gotten better since the conflict began in Feb 2014? | 1 = gotten worse  2 = stayed the same (**Skip to Q1e**)  3 = gotten better (**Skip to Q1e**) |  |
| 1d. | What is the **main** reason why you think it has gotten worse?  *Have the participant respond and match their answer to the options listed. Only one, main reason can be recorded* | 1 = loss of shelter  2 = shortage of water  3 = unable to afford food or poor quality of food available  4 = lack of income  5 = loss of transportation  6 = increased stress/anxiety  7 = preferred medication not available  8 = couldn’t access preferred provider  9 = natural progression of disease  10 = Other |  |
| 1e. | Since the conflict began in Feb 2014 were there times when you wanted to see a doctor or health care provider for your hypertension but could not? | 0 = No (**Skip to Q1g**)  1 = Yes |  |
| 1f. | Why do you think you were unable to see a doctor or a healthcare provider? | 1 = destruction of facilities/facilities not operational  2 = cost too expensive  3 = preferred providers not available  4 = lack of transportation  5 = other |  |
| 1g. | How many times in the last 3 months have you gone to see a doctor or health care provider about your hypertension? | 0 = none/no times  1 = 1 time  2 = 2 times  3 = 3 times  4 = >3 times  9 = Don’t know |  |
| 1h. | Have you been told to take medication for your hypertension? | 0 = No (**Skip to Section C2**)  1 = Yes  9 = Don’t know (**Skip to Section C2**) |  |
| 1i. | Are you currently taking medication for hypertension regularly/as prescribed? | 0 = No  1 = Yes (**Skip to Q1k**) |  |
| 1j. | What is the **main** reason why you are not taking your hypertension medication regularly? | 1 = preferred medication no longer available  2 = could not access pharmacy  3 = medication too expensive  4 = I don’t think I need it  5 = not currently prescribed  6 = other |  |
| 1k. | Since the conflict began in Feb 2014, have you experienced interruptions in your hypertension medication? | 0 = No (**Skip to Section C2**)  1 = Yes |  |
| 1l. | Why do you think you experienced interruptions in your hypertension medication? | 1 = preferred medication no longer available  2 = could not access pharmacy  3 = medication too expensive  4 = other |  |

2. Cardiovascular diseases (excluding hypertension)

| 2. | Have you been diagnosed with any cardiovascular diseases (excluding hypertension)?  *If say no at first, prompt by reading examples of CVD from 2a. If still answer no, skip to C3.* | 0 = No (**Skip to Section C3**)  1 = Yes |  | |
| --- | --- | --- | --- | --- |
| 2a. | What types of cardiovascular diseases?  *Select all that apply. Write numbers in box.* |  | | Yes |
|  |  | 1=Heart failure | |  |
|  |  | 2=Ischemic heart disease | |  |
|  |  | 3=Myocardial infarction | |  |
|  |  | 4=Stroke | |  |
|  |  | 5=Pre-stroke/pre-heart attack | |  |
|  |  | 5=Arrhythmia | |  |
|  |  | 6=Other | |  |
| 2aa. | Write number of most severe disease in box. All of the following questions relate to the most severe disease. |  |  | |
| 2b. | When were you diagnosed?  *Ask the participant to give you the date they were diagnosed. If Feb 2014 or before, select before. If after Feb 2014, select after.* | 0 = Before  1 = After |  | |
| 2c. | Has your CVD become worse, stayed the same, or gotten better since the conflict began in Feb 2014? | 1 = gotten worse  2 = stayed the same (**Skip to Q2e**)  3 = gotten better (**Skip to Q2e**) |  | |
| 2d. | What is the **main** reason why you think it has gotten worse?  *Have the participant respond and match their answer to the options listed. Only one, main reason can be recorded* | 1 = loss of shelter  2 = shortage of water  3 = unable to afford food or poor quality of food available  4 = lack of income  5 = loss of transportation  6 = increased stress/anxiety  7 = preferred medication not available  8 = couldn’t access preferred medical provider  9 = natural progression of disease  10 = Other |  | |
| 2e. | Since the conflict began in Feb 2014 were there times when you wanted to see a doctor or health care provider for your CVD but could not? | 0 = No (**Skip to Q2g**)  1 = Yes |  | |
| 2f. | Why do you think you were unable to see a doctor or a healthcare provider? | 1 = destruction of facilities/facilities not operational  2 = cost too expensive  3 = preferred providers not available  4 = lack of transportation  5 = other |  | |
| 2g. | How many times in the last 3 months have you gone to see a doctor or health care provider about your CVD? | 0 = none/no times  1 = 1 time  2 = 2 times  3 = 3 times  4 = >3 times  9 = Don’t know |  | |
| 2h. | Have you been told to take medication for your CVD? | 0 = No (**Skip to Section C3**)  1 = Yes  9 = Don’t know (**Skip to Section C3**) |  | |
| 2i. | Are you currently taking medication for CVD regularly/as prescribed? | 0 = No  1 = Yes (**Skip to Q2k**) |  | |
| 2j. | What is the **main** reason why you are not taking your CVD medication regularly? | 1 = preferred medication no longer available  2 = could not access pharmacy  3 = medication too expensive  4 = I don’t think I need it  5 = not currently prescribed  6 = other |  | |
| 2k. | Since the conflict began in Feb 2014, have you experienced interruptions in your CVD medication? | 0 = No (**Skip to Section C3**)  1 = Yes |  | |
| 2l. | What is the **main** reason why you experienced interruptions in your CVD medication? | 1 = preferred medication no longer available  2 = could not access pharmacy  3 = medication too expensive  4 = other |  | |

3. High cholesterol

| 3. | Have you been diagnosed with high blood cholesterol? | 0 = No (**Skip to Section C4**)  1 = Yes |  |
| --- | --- | --- | --- |
| 3b. | When were you diagnosed?  *Ask the participant to give you the date they were diagnosed. If Feb 2014 or before, select before. If after Feb 2014, select after.* | 0 = Before  1 = After |  |
| 3c. | Has your high cholesterol become worse, stayed the same, or gotten better since the conflict began in Feb 2014? | 1 = gotten worse  2 = stayed the same (**Skip to Q3e**)  3 = gotten better (**Skip to Q3e**) |  |
| 3d. | What is the **main** reason why you think it has gotten worse?  *Have the participant respond and match their answer to the options listed. Only one, main reason can be recorded* | 1 = loss of shelter  2 = shortage of water  3 = unable to afford food or poor quality of food available  4 = lack of income  5 = loss of transportation  6 = increased stress/anxiety  7 = preferred medication not available  8 = couldn’t access preferred provider  9 = natural progression of disease  10 = Other |  |
| 3e. | Since the conflict began in Feb 2014 were there times when you wanted to see a doctor or health care provider for your high cholesterol but could not? | 0 = No (**Skip to Q3g**)  1 = Yes |  |
| 3f. | Why do you think you were unable to see a doctor or a healthcare provider? | 1 = destruction of facilities/facilities not operational  2 = cost too expensive  3 = preferred providers not available  4 = lack of transportation  5 = other |  |
| 3g. | How many times in the last 3 months have you gone to see a doctor or health care provider about your high cholesterol? | 0 = none/no times  1 = 1 time  2 = 2 times  3 = 3 times  4 = >3 times  9 = Don’t know |  |
| 3h. | Have you been told to take medication for your high cholesterol? | 0 = No (**Skip to Section C4**)  1 = Yes  9 = Don’t know (**Skip to Section C4**) |  |
| 3i. | Are you currently taking medication for high cholesterol regularly/as prescribed? | 0 = No  1 = Yes (**Skip to Q3k**) |  |
| 3j. | What is the **main** reason why you are not taking your high cholesterol medication regularly? | 1 = preferred medication no longer available  2 = could not access pharmacy  3 = medication too expensive  4 = I don’t think I need it  5 = not currently prescribed  6 = other |  |
| 3k. | Since the conflict began in Feb 2014, have you experienced interruptions in your high cholesterol medication? | 0 = No (**Skip to Section C4**)  1 = Yes |  |
| 3l. | What is the **main** reason why you experienced interruptions in your high cholesterol medication? | 1 = preferred medication no longer available  2 = could not access pharmacy  3 = medication too expensive  4 = other |  |

4. Chronic lung disorders

| 4. | Have you been diagnosed with any chronic lung disorders?  *If say no at first, prompt by reading examples from 4a. If still answer no, skip to C5.* | 0 = No (**Skip to Section C5**)  1 = Yes |  | |
| --- | --- | --- | --- | --- |
| 4a. | What types of chronic lung disorders?  *Select all that apply* |  | | Yes |
|  |  | 1=Chronic obstructive pulmonary disorder (COPD) | |  |
|  |  | 2=Asthma | |  |
|  |  | 3=Other | |  |
| 4aa. | Write number of most severe disease in box. All of the following questions relate to the most severe disease. |  |  | |
| 4b. | When were you diagnosed?  *Ask the participant to give you the date they were diagnosed. If Feb 2014 or before, select before. If after Feb 2014, select after.* | 0 = Before  1 = After |  | |
| 4c. | Has your chronic lung disorder become worse, stayed the same, or gotten better since the conflict began in Feb 2014? | 1 = gotten worse  2 = stayed the same (**Skip to Q4e**)  3 = gotten better (**Skip to Q4e**) |  | |
| 4d. | What is the **main** reason why you think it has gotten worse?  *Have the participant respond and match their answer to the options listed. Only one, main reason can be recorded* | 1 = loss of shelter  2 = shortage of water  3 = unable to afford food or poor quality of food available  4 = lack of income  5 = loss of transportation  6 = increased stress/anxiety  7 = preferred medication not available  8 = couldn’t access preferred provider  9 = natural progression of disease  10 = Other |  | |
| 4e. | Since the conflict began in Feb 2014 were there times when you wanted to see a doctor or health care provider for your chronic lung disorder but could not? | 0 = No (**Skip to Q4g**)  1 = Yes |  | |
| 4f. | Why do you think you were unable to see a doctor or a healthcare provider? | 1 = destruction of facilities/facilities not operational  2 = cost too expensive  3 = preferred providers not available  4 = lack of transportation  5 = other |  | |
| 4g. | How many times in the last 3 months have you gone to see a doctor or health care provider about your chronic lung disorder? | 0 = none/no times  1 = 1 time  2 = 2 times  3 = 3 times  4 = >3 times  9 = Don’t know |  | |
| 4h. | Have you been told to take medication for your chronic lung disorder? | 0 = No (**Skip to Section C5**)  1 = Yes  9 = Don’t know (**Skip to Section C5**) |  | |
| 4i. | Are you currently taking medication for your chronic lung disorder regularly/as prescribed? | 0 = No  1 = Yes (**Skip to next Q4k**) |  | |
| 4j. | What is the **main** reason why you are not taking your chronic lung disorder medication regularly? | 1 = preferred medication no longer available  2 = could not access pharmacy  3 = medication too expensive  4 = I don’t think I need it  5 = not currently prescribed  6 = other |  | |
| 4k. | Since the conflict began in Feb 2014, have you experienced interruptions in your chronic lung disorder medication? | 0 = No (**Skip to Section C5**)  1 = Yes |  | |
| 4l. | What is the **main** reason why you experienced interruptions in your chronic lung disorder medication? | 1 = preferred medication no longer available  2 = could not access pharmacy  3 = medication too expensive  4 = other |  | |

5. Diabetes

| 5. | Have you been diagnosed with diabetes or prediabetes?  *If say no at first, prompt by reading examples from5a. If still answer no, skip to C6.* | 0 = No **(Skip to Section C6)**  1 = Yes |  |
| --- | --- | --- | --- |
| 5a. | What type of diabetes or prediabetes?  *Select which type* |  | Yes |
|  |  | Type 1 diabetes |  |
|  |  | Type 2 diabetes |  |
|  |  | Pre-diabetes |  |
|  |  | Gestational diabetes |  |
| 5b. | When were you diagnosed?  *Ask the participant to give you the date they were diagnosed. If Feb 2014 or before, select before. If after Feb 2014, select after.* | 0 = Before  1 = After |  |
| 5c. | Has your diabetes become worse, stayed the same, or gotten better since the conflict began in Feb 2014? | 1 = gotten worse  2 = stayed the same (**Skip to Q5e**)  3 = gotten better (**Skip to Q5e**) |  |
| 5d. | What is the **main** reason why you think it has gotten worse?  *Have the participant respond and match their answer to the options listed. Only one, main reason can be recorded* | 1 = loss of shelter  2 = shortage of water  3 = unable to afford food or poor quality of food available  4 = lack of income  5 = loss of transportation  6 = increased stress/anxiety  7 = preferred medication not available  8 = couldn’t access preferred provider  9 = natural progression of disease  10 = Other |  |
| 5e. | Since the conflict began in Feb 2014 were there times when you wanted to see a doctor or health care provider for your diabetes but could not? | 0 = No (**Skip to Q5g**)  1 = Yes |  |
| 5f. | Why do you think you were unable to see a doctor or a healthcare provider? | 1 = destruction of facilities/facilities not operational  2 = cost too expensive  3 = preferred providers not available  4 = lack of transportation  5 = other |  |
| 5g. | How many times in the last 3 months have you gone to see a doctor or health care provider about your diabetes? | 0 = none/no times  1 = 1 time  2 = 2 times  3 = 3 times  4 = >3 times  9 = Don’t know |  |
| 5h. | Have you been told to take medication for your diabetes? | 0 = No (**Skip to Section C6**)  1 = Yes  9 = Don’t know (**Skip to Section C6**) |  |
| 5i. | Are you currently taking medication for diabetes regularly/as prescribed? | 0 = No  1 = Yes (**Skip to Q5k**) |  |
| 5j. | What is the **main** reason why you are not taking your diabetes medication regularly? | 1 = preferred medication no longer available  2 = could not access pharmacy  3 = medication too expensive  4 = I don’t think I need it  5 = not currently prescribed  6 = other |  |
| 5k. | Since the conflict began in Feb 2014, have you experienced interruptions in your diabetes medication? | 0 = No (**Skip to Section C6**)  1 = Yes |  |
| 5l. | What is the **main** reason why you experienced interruptions in your diabetes medication? | 1 = preferred medication no longer available  2 = could not access pharmacy  3 = medication too expensive  4 = other |  |

6 Cancers

| 6. | Have you been diagnosed with any cancers?  *If say no at first, prompt by reading examples from 6a. If still answer no, skip to C7.* | 0 = No (**Skip to Section C7**)  1 = Yes |  | |
| --- | --- | --- | --- | --- |
| 6a. | What type(s) of cancer?  *Select from the following* |  | | Yes |
|  |  | 1=Colorectal cancer | |  |
|  |  | 2=Cancer of the reproductive organs or breast cancer | |  |
|  |  | 3=Lung cancer | |  |
|  |  | 4=Prostate cancer | |  |
|  |  | 5=Other | |  |
| 6aa. | Write number of most severe disease in box. All of the following questions relate to the most severe disease. |  |  | |
| 6b. | When were you diagnosed?  *Ask the participant to give you the date they were diagnosed. If before Feb 2014, select before. If after Feb 2014, select after.* | 0 = Before  1 = After |  | |
| 6c. | Has your cancer become worse, stayed the same, or gotten better since the conflict began in Feb 2014? | 1 = gotten worse  2 = stayed the same (**Skip to Q6e**)  3 = gotten better (**Skip to Q6e**) |  | |
| 6d. | What is the **main** reason why you think it has gotten worse?  *Have the participant respond and match their answer to the options listed. Only one,* ***main*** *reason can be recorded* | 1 = loss of shelter  2 = shortage of water  3 = unable to afford food or poor quality of food available  4 = lack of income  5 = loss of transportation  6 = increased stress/anxiety  7 = preferred medication not available  8 = couldn’t access preferred medical provider  9 = natural progression of disease  10 = Other |  | |
| 6e. | Since the conflict began in Feb 2014 were there times when you wanted to see a doctor or health care provider for your cancer but could not? | 0 = No (**Skip to Q6g**)  1 = Yes |  | |
| 6f. | Why do you think you were unable to see a doctor or a healthcare provider? | 1 = destruction of facilities/facilities not operational  2 = cost too expensive  3 = preferred providers not available  4 = lack of transportation  5 = other |  | |
| 6g. | How many times in the last 3 months have you gone to see a doctor or health care provider about your cancer? | 0 = none/no times  1 = 1 time  2 = 2 times  3 = 3 times  4 = >3 times  9 = Don’t know |  | |
| 6h. | Have you been told to take medication for your cancer? | 0 = No (**Skip to Section C7**)  1 = Yes  9 = Don’t know (**Skip to Section C7**) |  | |
| 6i. | Are you currently taking medication for cancer regularly/as prescribed? | 0 = No  1 = Yes (**Skip to Q6k**) |  | |
| 6j. | What is the **main** reason why you are not taking your cancer medication regularly? | 1 = preferred medication no longer available  2 = could not access pharmacy  3 = medication too expensive  4 = I don’t think I need it  5 = not currently prescribed  6 = other |  | |
| 6k. | Since the conflict began in Feb 2014, have you experienced interruptions in your cancer medication? | 0 = No (**Skip to Section C7**)  1 = Yes |  | |
| 6l. | What is the **main** reason why you experienced interruptions in your cancer medication? | 1 = preferred medication no longer available  2 = could not access pharmacy  3 = medication too expensive  4 = other |  | |

7. Tuberculosis

| 7. | Have you been diagnosed with tuberculosis or TB? | 0 = No (**Skip to Section C8)**  1 = Yes |  |
| --- | --- | --- | --- |
| 7b. | When were you diagnosed?  *Ask the participant to give you the date they were diagnosed. If Feb 2014 or before, select before. If after Feb 2014, select after.* | 0 = Before  1 = After |  |
| 7c. | Has your TB become worse, stayed the same, or gotten better since the conflict began in Feb 2014? | 1 = gotten worse  2 = stayed the same (**Skip to Q7e**)  3 = gotten better (**Skip to Q7e**) |  |
| 7d. | What is the **main** reason why you think it has gotten worse?  *Have the participant respond and match their answer to the options listed. Only one, main reason can be recorded* | 1 = loss of shelter  2 = shortage of water  3 = unable to afford food or poor quality of food available  4 = lack of income  5 = loss of transportation  6 = increased stress/anxiety  7 = preferred medication not available  8 = couldn’t access preferred provider  9 = natural progression of disease  10 = Other |  |
| 7e. | Since the conflict began in Feb 2014 were there times when you wanted to see a doctor or health care provider for your TB but could not? | 0 = No (**Skip to Q7g**)  1 = Yes |  |
| 7f. | Why do you think you were unable to see a doctor or a healthcare provider? | 1 = destruction of facilities/facilities not operational  2 = cost too expensive  3 = preferred providers not available  4 = lack of transportation  5 = other |  |
| 7g. | How many times in the last 3 months have you gone to see a doctor or health care provider about your TB? | 0 = none/no times  1 = 1 time  2 = 2 times  3 = 3 times  4 = >3 times  9 = Don’t know |  |
| 7h. | Have you been told to take medication for your TB? | 0 = No (**Skip to Section C8**)  1 = Yes  9 = Don’t know (**Skip to Section C8**) |  |
| 7i. | Are you currently taking medication for TB regularly/as prescribed? | 0 = No  1 = Yes (**Skip to Q7k**) |  |
| 7j. | What is the **main** reason why you are not taking your TB medication regularly? | 1 = preferred medication no longer available  2 = could not access pharmacy  3 = medication too expensive  4 = I don’t think I need it  5 = not currently prescribed  6 = other |  |
| 7k. | Since the conflict began in Feb 2014, have you experienced interruptions in your TB medication? | 0 = No (**Skip to Section C8**)  1 = Yes |  |
| 7l. | What is the **main** reason why you experienced interruptions in your TB medication? | 1 = preferred medication no longer available  2 = could not access pharmacy  3 = medication too expensive  4 = other |  |

8. Mental illness

| 8. | Have you been diagnosed with any mental illness?  *If say no at first, prompt by reading examples from 8a. If still answer no, skip to C9.* | 0 = No (**Skip to Section C9**)  1 = Yes |  | |
| --- | --- | --- | --- | --- |
| 8a. | Which mental illness?  *Select all that apply* |  | | Yes |
|  |  | 1=Depression | |  |
|  |  | 2=Schizophrenia | |  |
|  |  | 3=Post-traumatic stress disorder (PTSD) | |  |
|  |  | 4=Anxiety | |  |
|  |  | 5=Manic depression (bipolar disorder) | |  |
|  |  | 6=Other | |  |
| 8aa. | Write number of most severe disease in box. All of the following questions relate to the most severe disease. |  |  | |
| 8b. | When were you diagnosed?  *Ask the participant to give you the date they were diagnosed. If Feb 2014 or before, select before. If after Feb 2014, select after.* | 0 = Before  1 = After |  | |
| 8c. | Has your mental illness become worse, stayed the same, or gotten better since the conflict began in Feb 2014? | 1 = gotten worse  2 = stayed the same (**Skip to Q8e**)  3 = gotten better (**Skip to Q8e**) |  | |
| 8d. | What is the **main** reason why you think it has gotten worse?  *Have the participant respond and match their answer to the options listed. Only one, main reason can be recorded* | 1 = loss of shelter  2 = shortage of water  3 = unable to afford food or poor quality of food available  4 = lack of income  5 = loss of transportation  6 = increased stress/anxiety  7 = preferred medication not available  8 = couldn’t access preferred provider  9 = natural progression of disease  10 = Other |  | |
| 8e. | Since the conflict began in Feb 2014 were there times when you wanted to see a doctor or health care provider for your mental illness but could not? | 0 = No (**Skip to Q8g**)  1 = Yes |  | |
| 8f. | Why do you think you were unable to see a doctor or a healthcare provider? | 1 = destruction of facilities/facilities not operational  2 = cost too expensive  3 = preferred providers not available  4 = lack of transportation  5 = other |  | |
| 8g. | How many times in the last 3 months have you gone to see a doctor or health care provider about your mental illness? | 0 = none/no times  1 = 1 time  2 = 2 times  3 = 3 times  4 = >3 times  9 = Don’t know |  | |
| 8h. | Have you been told to take medication for your mental illness? | 0 = No (**skip to Section C9**)  1 = Yes  9 = Don’t know (**skip to Section C9**) |  | |
| 8i. | Are you currently taking medication for your mental illness regularly/as prescribed? | 0 = No  1 = Yes (**Skip to Q8k**) |  | |
| 8j. | What is the **main** reason why you are not taking your medication for your mental illness regularly? | 1 = preferred medication no longer available  2 = could not access pharmacy  3 = medication too expensive  4 = I don’t think I need it  5 = not currently prescribed  6 = other |  | |
| 8k. | Since the conflict began in Feb 2014, have you experienced interruptions in your mental illness medication? | 0 = No (**skip to Section C9**)  1 = Yes |  | |
| 8l. | What is the **main** reason why you experienced interruptions in your mental illness medication? | 1 = preferred medication no longer available  2 = could not access pharmacy  3 = medication too expensive  4 = other |  | |

9. Other chronic conditions

| 9. | Have you been diagnosed with any other chronic conditions? | 0 = No  1 = Yes |  |
| --- | --- | --- | --- |
| 9a. | Which chronic condition of those you have is the most important or severe (not counting those already asked and answered)?  *Have the participant to list all other chronic conditions, then ask* |  |  |
| 2aa. | Write name of most severe disease on the line. All of the following questions relate to the most severe disease. |  | ____________________ |
| 9b. | When were you diagnosed?  *Ask the participant to give you the date they were diagnosed. If Feb 2014 or before, select before. If after Feb 2014, select after.* | 0 = Before  1 = After |  |
| 9c. | Has your condition become worse, stayed the same, or gotten better since the conflict began in Feb 2014? | 1 = gotten worse  2 = stayed the same (**Skip to Q9e**)  3 = gotten better (**Skip to Q9e**) |  |
| 9d. | What is the **main** reason why you think it has gotten worse?  *Have the participant respond and match their answer to the options listed. Only one, main reason can be recorded* | 1 = loss of shelter  2 = shortage of water  3 = unable to afford food or poor quality of food available  4 = lack of income  5 = loss of transportation  6 = increased stress/anxiety  7 = preferred medication not available  8 = couldn’t access preferred provider  9 = natural progression of disease  10 = Other |  |
| 9e. | Since the conflict began in Feb 2014 were there times when you wanted to see a doctor or health care provider for your condition but could not? | 0 = No (**Skip to Q9g**)  1 = Yes |  |
| 9f. | Why do you think you were unable to see a doctor or a healthcare provider? | 1 = destruction of facilities/facilities not operational  2 = cost too expensive  3 = preferred providers not available  4 = lack of transportation  5 = other |  |
| 9g. | How many times in the last 3 months have you gone to see a doctor or health care provider about your condition? | 0 = none/no times  1 = 1 time  2 = 2 times  3 = 3 times  4 = >3 times  9 = Don’t know |  |
| 9h. | Have you been told to take medication for your condition? | 0 = No (**Skip to Section D**)  1 = Yes  9 = Don’t know (**Skip to Section D**) |  |
| 9i. | Are you currently taking medication for your condition regularly/as prescribed? | 0 = No  1 = Yes (**Skip to Q9k**) |  |
| 9j. | What is the **main** reason why you are not taking your medication regularly? | 1 = preferred medication no longer available  2 = could not access pharmacy  3 = medication too expensive  4 = I don’t think I need it  5 = not currently prescribed  6 = other |  |
| 9k. | Since the conflict began in Feb 2014, have you experienced interruptions in your medication? | 0 = No (**Skip to Section D**)  1 = Yes |  |
| 9l. | What is the **main** reason why you experienced interruptions in your medication? | 1 = preferred medication no longer available  2 = could not access pharmacy  3 = medication too expensive  4 = other |  |

**Section D. Weight status**

| 1. | What is your height (cm)? | Write height in cm | |
| --- | --- | --- | --- |
| 2. | What is your weight (kg)? | Write weight in kg | |
| 3. | Have you been told by a doctor that you are overweight or obese? | 0=No (**Skip to Section E**)  1= Yes  9= Don’t know (**Skip to Section E**) |  |
| 4. | Have you been told to take medication to lose weight? | 0 = No (**Skip to Q7**)  1 = Yes  9 = Don’t Know (**Skip to Q7**) |  |
| 5. | Are you taking medication to lose weight regularly? | 0 = No  1 = Yes (**Skip to Q7**)  9 = Don’t Know (**Skip to Q7**) |  |
| 6. | What is the **main** reason why you are not taking your weight medication regularly/as prescribed? | 1 = Can’t access pharmacy (it is far)  2 = Medication not available at pharmacy  3 = Medication is too expensive  4 = Other  9 = Don’t Know |  |
| 7. | Have you been told by doctor to go on a diet to lose weight? | 0 = No (**Skip to Section E**)  1 = Yes  9 = Don’t Know (**Skip to Section E**) |  |
| 8. | Are you following the prescribed diet regularly? | 0 = No  1 = Yes (**Skip to Section E**)  9 = Don’t Know (**Skip to Section E**) |  |
| 9. | What is the **main** reason why you are not following the prescribed diet regularly? | 1 = Recommended food/products not available in markets  2 = Recommended food/products too expensive  3 = I decided not to do it myself  4 = Other  9 = Don’t Know |  |

STOP HERE IF ANSWERING BY PROXY

**Section E: Psychological Condition (Kessler -6)**

*Instructions:* ***SAY*** ***“Now I will ask you questions about how you have been feeling in the past 30 days. Please select one answer per question.”***

| **1.** | During the past 30 days, about how often did you feel nervous? Would you say all of the time, most of the time, some of the time, a little of the time, or none of the time? | 0 = None of the time  1 = A little of the time  2 = Some of the time  3 = Most of the time  4 = All of the time  6 = Refused  9 = Don’t know |  |
| --- | --- | --- | --- |
| **2.** | During the past 30 days, about how often did you feel hopeless? Would you say all of the time, most of the time, some of the time, a little of the time, or none of the time? | 0 = None of the time  1 = A little of the time  2 = Some of the time  3 = Most of the time  4 = All of the time  6 = Refused  9 = Don’t know |  |
| **3.** | During the past 30 days, about how often did you feel restless or fidgety? Would you say all of the time, most of the time, some of the time, a little of the time, or none of the time? | 0 = None of the time  1 = A little of the time  2 = Some of the time  3 = Most of the time  4 = All of the time  6 = Refused  9 = Don’t know |  |
| **4.** | During the past 30 days, about how often did you feel so depressed that nothing could cheer you up? Would you say all of the time, most of the time, some of the time, a little of the time, or none of the time? | 0 = None of the time  1 = A little of the time  2 = Some of the time  3 = Most of the time  4 = All of the time  6 = Refused  9 = Don’t know |  |
| **5.** | During the past 30 days, about how often did you feel that everything was an effort? Would you say all of the time, most of the time, some of the time, a little of the time, or none of the time? | 0 = None of the time  1 = A little of the time  2 = Some of the time  3 = Most of the time  4 = All of the time  6 = Refused  9 = Don’t know |  |
| **6.** | During the past 30 days, about how often did you feel worthless? Would you say all of the time, most of the time, some of the time, a little of the time, or none of the time? | 0 = None of the time  1 = A little of the time  2 = Some of the time  3 = Most of the time  4 = All of the time  6 = Refused  9 = Don’t know |  |

**Section F: Traumatic events exposure**

*Instructions:* ***SAY*** ***“Now I’m going to ask you about things that might have happened since the violence began in February 2014.”*** *Select one answer per question*

| **1.** | Lack of food or water | 0 = No  1 = Experienced  2 = Witnessed  3 = Heard about  6 = Refused  9 = Don’t know |  |
| --- | --- | --- | --- |
| **2.** | Lack of shelter | 0 = No  1 = Experienced  2 = Witnessed  3 = Heard about  6 = Refused  9 = Don’t know |  |
| **3.** | Ill health without access to medical care | 0 = No  1 = Experienced  2 = Witnessed  3 = Heard about  6 = Refused  9 = Don’t know |  |
| **4.** | Loss or destruction of property or belongings | 0 = No  1 = Experienced  2 = Witnessed  3 = Heard about  6 = Refused  9 = Don’t know |  |
| **5.** | Forced displacement | 0 = No  1 = Experienced  2 = Witnessed  3 = Heard about  6 = Refused  9 = Don’t know |  |
| **6.** | Forced separation from family members | 0 = No  1 = Experienced  2 = Witnessed  3 = Heard about  6 = Refused  9 = Don’t know |  |
| **7.** | Missing or lost family member(s) | 0 = No  1 = Experienced  2 = Witnessed  3 = Heard about  6 = Refused  9 = Don’t know |  |
| **8.** | Living in collective centers | 0 = No  1 = Experienced  2 = Witnessed  3 = Heard about  6 = Refused  9 = Don’t know |  |
| **9.** | Kidnapping or abduction | 0 = No  1 = Experienced  2 = Witnessed  3 = Heard about  6 = Refused  9 = Don’t know |  |
| **10.** | Imprisonment | 0 = No  1 = Experienced  2 = Witnessed  3 = Heard about  6 = Refused  9 = Don’t know |  |
| **11.** | Interrogation/harassment by combatants and/or others with threats to life | 0 = No  1 = Experienced  2 = Witnessed  3 = Heard about  6 = Refused  9 = Don’t know |  |
| **12.** | Beatings by combatants and/or others | 0 = No  1 = Experienced  2 = Witnessed  3 = Heard about  6 = Refused  9 = Don’t know |  |
| **13.** | Attacks/bombardment with shells/rockets | 0 = No  1 = Experienced  2 = Witnessed  3 = Heard about  6 = Refused  9 = Don’t know |  |
| **14.** | Shot at with a gun | 0 = No  1 = Experienced  2 = Witnessed  3 = Heard about  6 = Refused  9 = Don’t know |  |
| **15.** | Injury from a knife, gun, or other weapon | 0 = No  1 = Experienced  2 = Witnessed  3 = Heard about  6 = Refused  9 = Don’t know |  |
| **16.** | Injury from a landmine or UXO | 0 = No  1 = Experienced  2 = Witnessed  3 = Heard about  6 = Refused  9 = Don’t know |  |
| **17.** | Rape or sexual abuse | 0 = No  1 = Experienced  2 = Witnessed  3 = Heard about  6 = Refused  9 = Don’t know |  |
| **18.** | Disability from an injury received during a battle or attack | 0 = No  1 = Experienced  2 = Witnessed  3 = Heard about  6 = Refused  9 = Don’t know |  |
| **19.** | Caught in the crossfire of an attack or battle | 0 = No  1 = Experienced  2 = Witnessed  3 = Heard about  6 = Refused  9 = Don’t know |  |
| **20.** | Death of a family member or friend due to illness or lack of food | 0 = No  2 = Witnessed  3 = Heard about  6 = Refused  9 = Don’t know |  |
| **21.** | Death of a family member due to fighting or murder | 0 = No  2 = Witnessed  3 = Heard about  6 = Refused  9 = Don’t know |  |
| **22.** | Death of other acquaintance due to fighting or murder | 0 = No  2 = Witnessed  3 = Heard about  6 = Refused  9 = Don’t know |  |

**Section G: PTSD Symptoms (Harvard Trauma Questionnaire)**

*Instructions:* ***SAY*** ***“In the past 30 days have you experienced any of the following?”*** Select only one answer per question

| **1.** | Recurrent thoughts or memories of the most hurtful or terrifying events | 0 = Not at all  1 = A little  2 = Quite a bit  3 = Extremely  6 = Refused  9 = Don’t know |  |
| --- | --- | --- | --- |
| **2.** | Feeling as though the hurtful or terrifying event is happening again | 0 = Not at all  1 = A little  2 = Quite a bit  3 = Extremely  6 = Refused  9 = Don’t know |  |
| **3.** | Nightmares | 0 = Not at all  1 = A little  2 = Quite a bit  3 = Extremely  6 = Refused  9 = Don’t know |  |
| **4.** | Feeling detached or withdrawn from people | 0 = Not at all  1 = A little  2 = Quite a bit  3 = Extremely  6 = Refused  9 = Don’t know |  |
| **5.** | Unable to feel emotions | 0 = Not at all  1 = A little  2 = Quite a bit  3 = Extremely  6 = Refused  9 = Don’t know |  |
| **6.** | Feeling jumpy, easily startled | 0 = Not at all  1 = A little  2 = Quite a bit  3 = Extremely  6 = Refused  9 = Don’t know |  |
| **7.** | Difficulty concentrating | 0 = Not at all  1 = A little  2 = Quite a bit  3 = Extremely  6 = Refused  9 = Don’t know |  |
| **8.** | Trouble sleeping | 0 = Not at all  1 = A little  2 = Quite a bit  3 = Extremely  6 = Refused  9 = Don’t know |  |
| **9.** | Feeling on guard | 0 = Not at all  1 = A little  2 = Quite a bit  3 = Extremely  6 = Refused  9 = Don’t know |  |
| **10.** | Feeling irritable or having outburst of anger | 0 = Not at all  1 = A little  2 = Quite a bit  3 = Extremely  6 = Refused  9 = Don’t know |  |
| **11.** | Avoiding activities that remind you of the traumatic or hurtful event | 0 = Not at all  1 = A little  2 = Quite a bit  3 = Extremely  6 = Refused  9 = Don’t know |  |
| **12.** | Inability to remember parts of the most traumatic or hurtful events | 0 = Not at all  1 = A little  2 = Quite a bit  3 = Extremely  6 = Refused  9 = Don’t know |  |
| **13.** | Less interest in daily activities | 0 = Not at all  1 = A little  2 = Quite a bit  3 = Extremely  6 = Refused  9 = Don’t know |  |
| **14.** | Feeling as if you don’t have a future | 0 = Not at all  1 = A little  2 = Quite a bit  3 = Extremely  6 = Refused  9 = Don’t know |  |
| **15.** | Avoiding thoughts or feelings associated with the traumatic or hurtful events | 0 = Not at all  1 = A little  2 = Quite a bit  3 = Extremely  6 = Refused  9 = Don’t know |  |
| **16.** | Sudden emotional or physical reaction when reminded of the most hurtful or traumatic events | 0 = Not at all  1 = A little  2 = Quite a bit  3 = Extremely  6 = Refused  9 = Don’t know |  |
| **17.** | Feeling guilty | 0 = Not at all  1 = A little  2 = Quite a bit  3 = Extremely  6 = Refused  9 = Don’t know |  |

**HH**

**MM**

**Time Visit Completed**
